# Supplementary material for: Identification of an amphipathic peptide sensor of the Bacillus subtilis fluid membrane microdomains
Source: Commun Biol. 2019 Aug 20;2:316. doi: 10.1038/s42003-019-0562-8 (PMC6702220; doi:10.1038/s42003-019-0562-8)
Supplement: Supplementary file 1 — Supplementary Information [file 42003_2019_562_MOESM1_ESM.pdf]

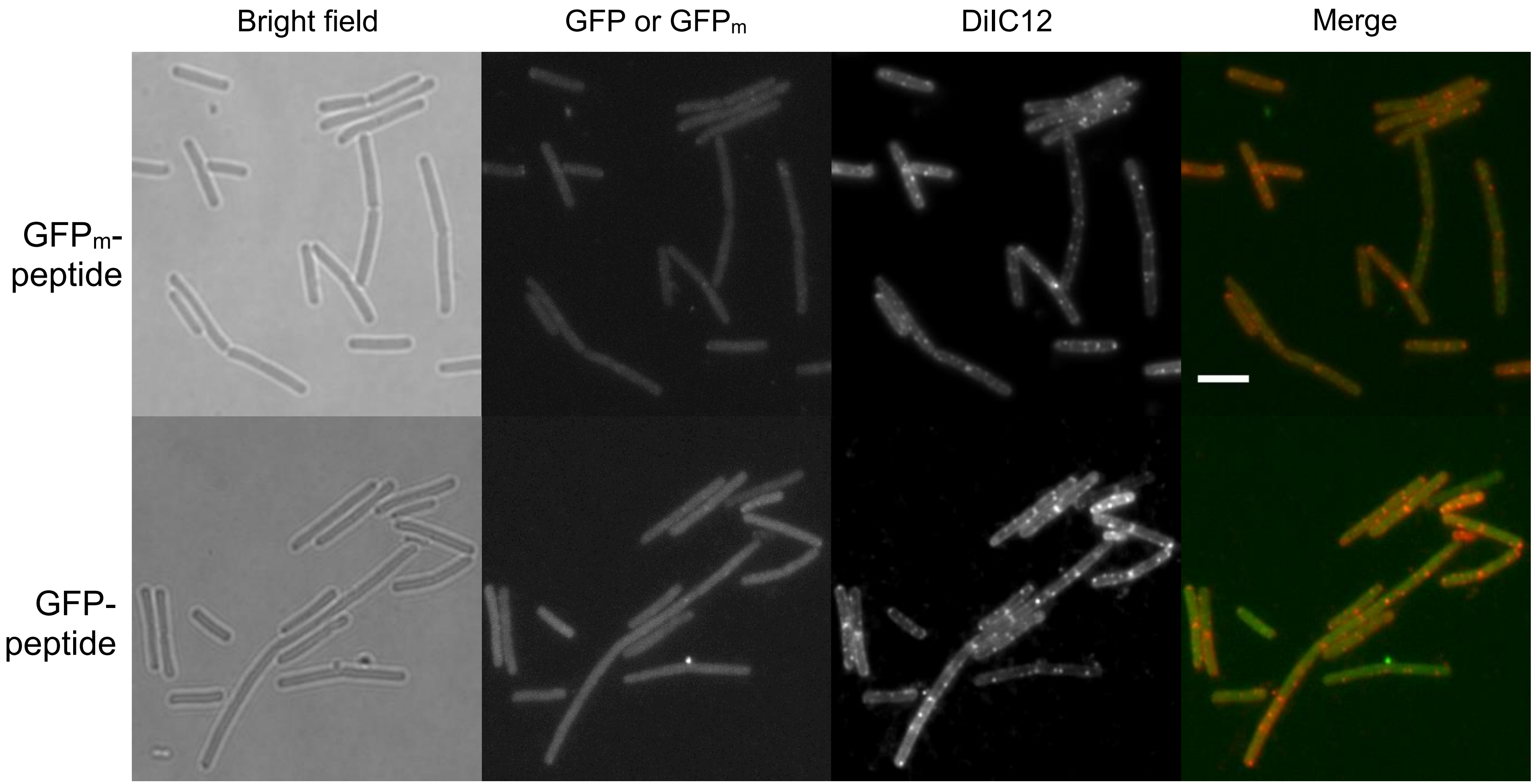

Supplementary Figure 1. No RIFs localization for a monomeric single amphipathic α-peptide. The α-peptide containing the residues 250-262 was fused to the C-terminus of the green fluorescent protein with (GFP_m) or without (GFP) the A206K mutation. The fusion protein was expressed in *Bacillus subtilis* and the cells were stained with DiIC12 and imaged as described in the Materials and Methods. Only one of the two C-terminally fused amphipathic α-peptides on a GFP dimer can interact with membrane, because dimeric GFP is known to have its two C-termini widely separated and located at opposite sides of the dimer interface [Suzuki et al. (2016) A novel mode of ubiquitin recognition by the ubiquitin-binding zinc finger domain of WRNIP1. *FEBS J.* 283: 2001-2017.]. The scale bar is 5 μm.

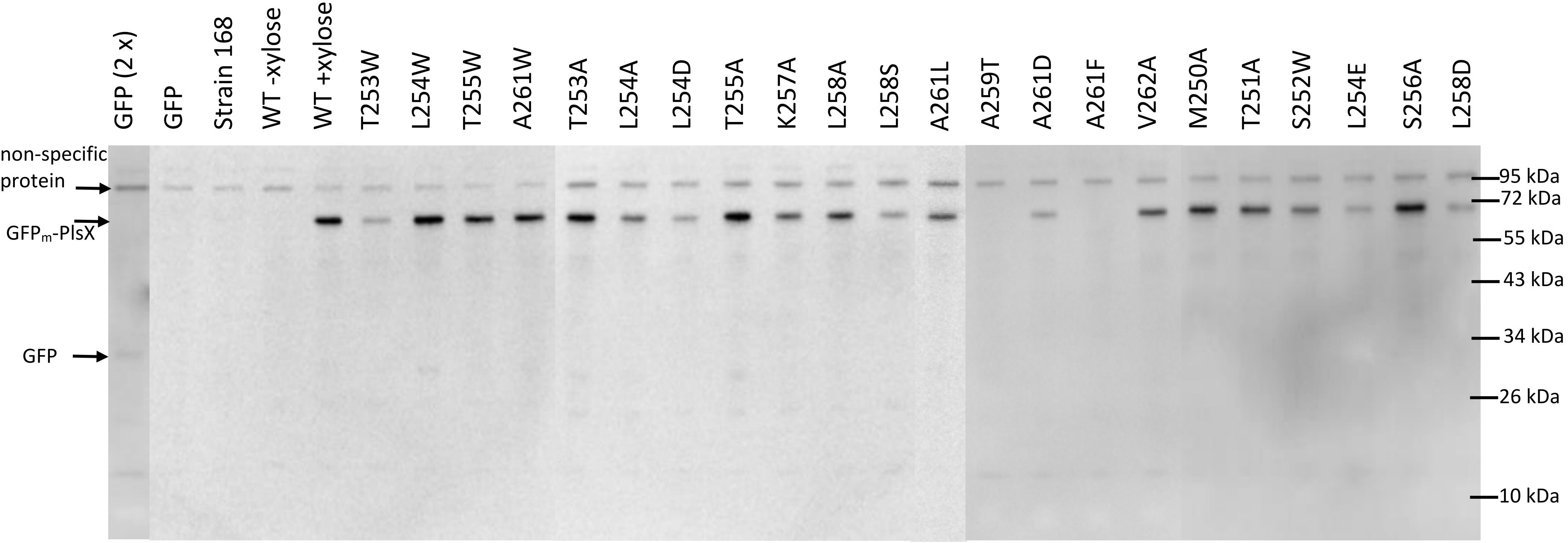

Supplementary Figure 2. Expression of GFP_m-PlsX and its mutants in *Bacillus subtilis* by Western blotting. GFP: a negative control using *Bacillus subtilis* cells transformed with the modified pSG1729 vector with doubled loading denoted by (2 ×); Strain 168: a negative control using culture of the *Bacillus subtilis* strain 168; WT-xylose and WT+xylose: positive controls using *Bacillus subtilis* cells expressing the wild-type GFP_m-PlsX in the absence and presence of 0.5% xylose, respectively. Mutants of GFP_m-PlsX are denoted by the mutations in the *plsX* gene.

Supplementary Figure 3

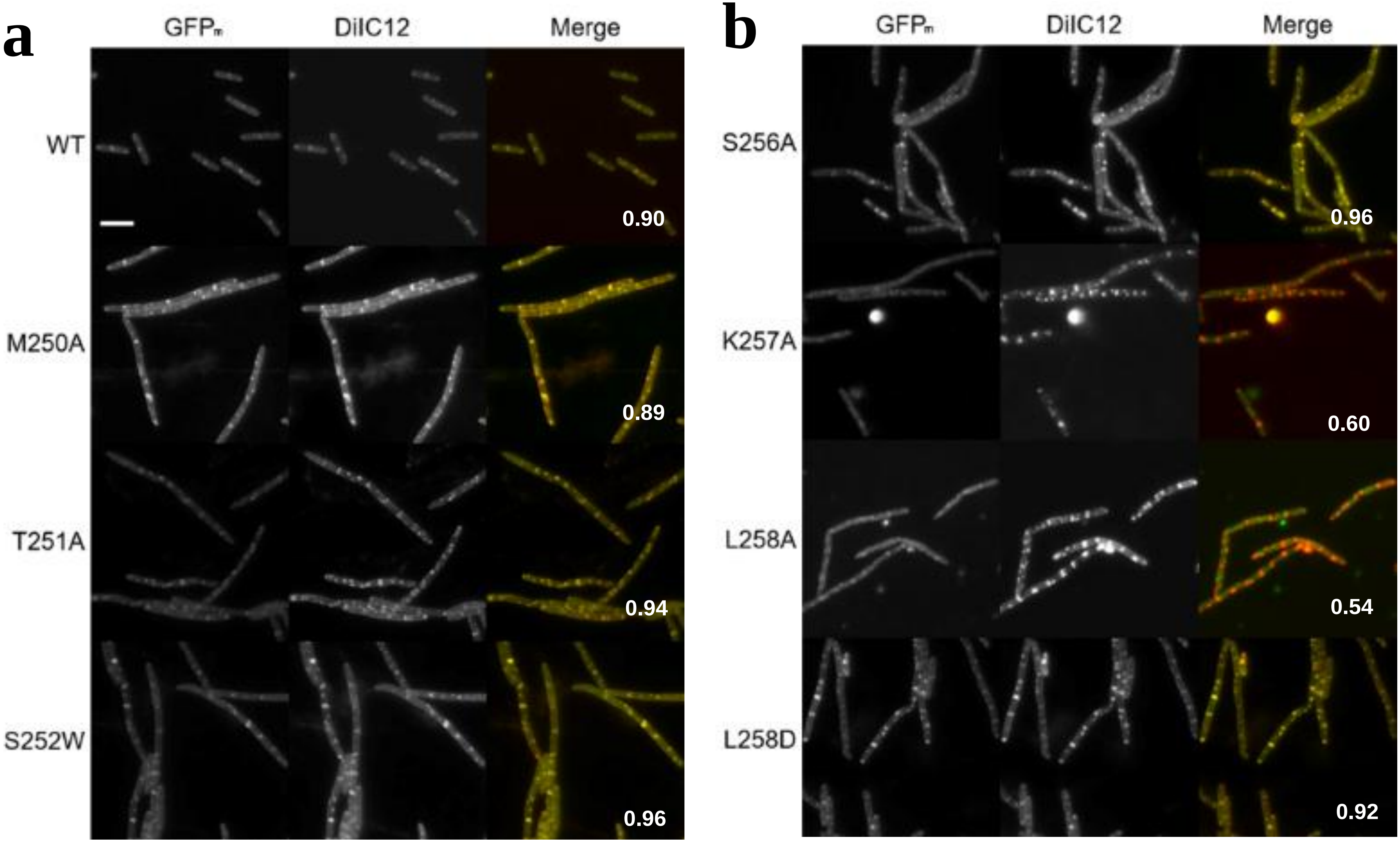

Supplementary Figure 3

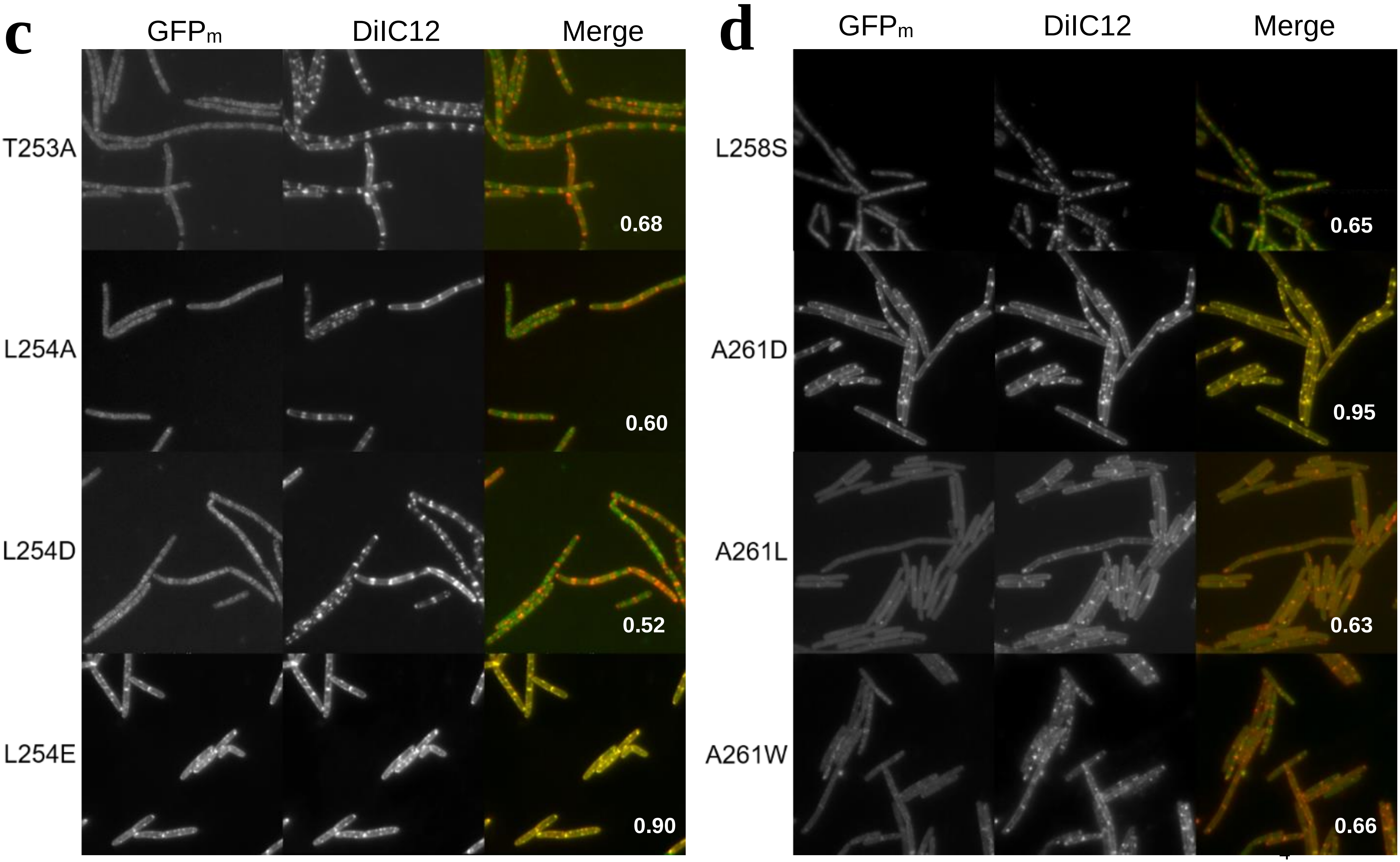

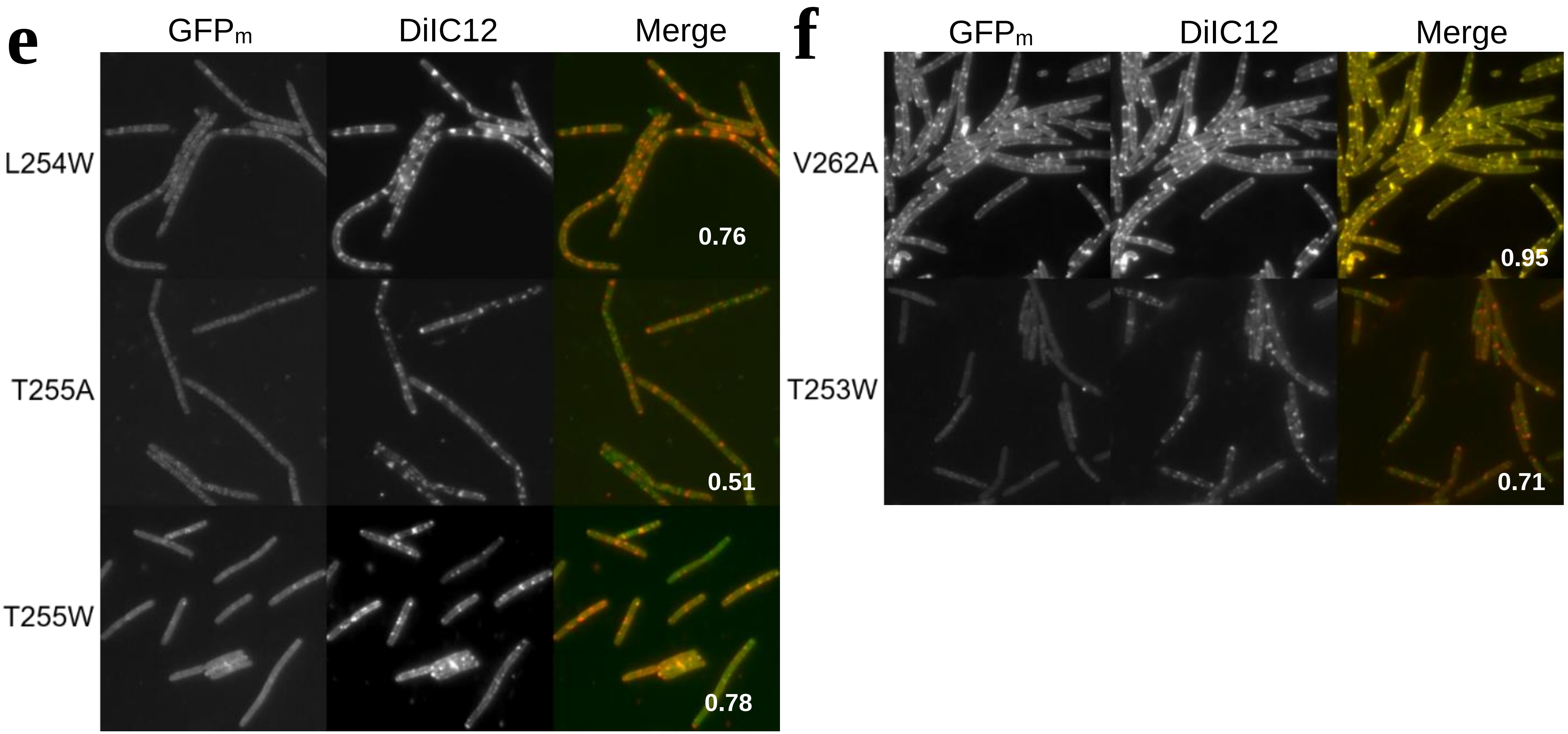

Supplementary Figure 3. Co-localization of GFP_m-PlsX and its mutants with the DiIC12-stained RIFs. Panels **a-f** include the cell images used to calculate the Pearson's correlation coefficient (R_r) as indicated in the merged images. Images presented Figure 2 were cut from the corresponding images shown here. The corresponding bright-field images are omitted for simplicity. The scale bar in the images of the wild-type fusion protein (WT) is 5 μm and applicable to all images.

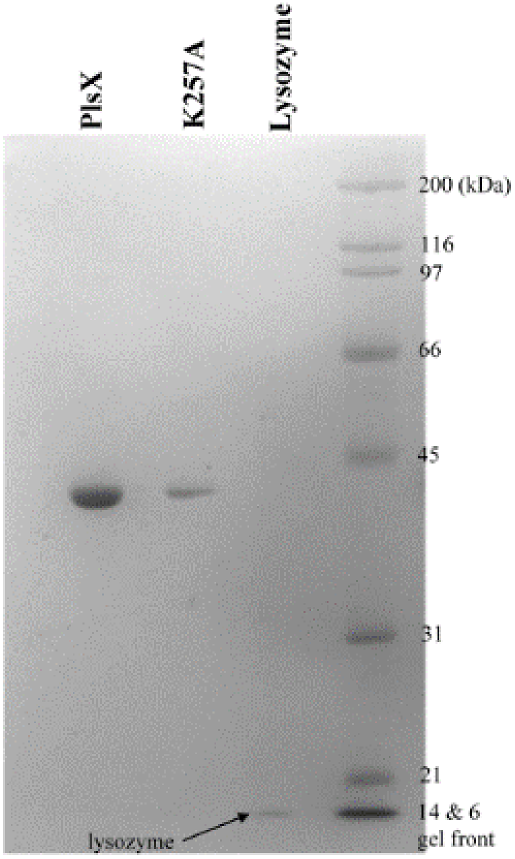

Supplementary Figure 4. Direct binding of PlsX to protein-free lipids of *Bacillus subtilis*. As a negative control, lysozyme was negligibly bound to the liposomes, while PlsX was significantly bound to the lipids. When the interaction was impaired by the K257A mutation, the amount of protein bound to the liposomes greatly decreased. These results provide strong support for the direct binding interaction between PlsX and the lipids. Protein-free total lipids were isolated from *Bacillus subtilis* and used to make liposome as described in 'Materials and Methods'. The tested proteins were incubated with the protein-free liposomes at a concentration of 2 mg/ml for 1 h at room temperature. The liposomes were separated from the supernatant by ultracentrifugation at 200,000 × g for 1 h and re-suspended in buffer for analysis by SDS-PAGE.

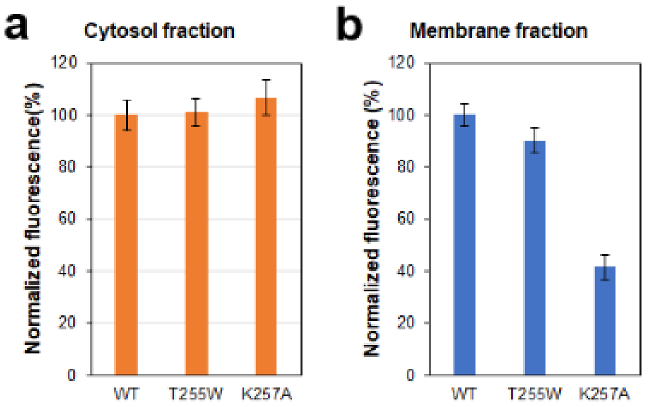

Supplementary Figure 5. Different partition of PlsX mutants between plasma membrane and cytosol. **a.** The GFP_m-fused protein in cytosol. **b.** The GFP_m-fused protein associated with the membrane. WT: wild-type GFP_m-PlsX; T255W: GFP_m-T255W; and T257A: GFP_m-T257A. *Bacillus subtilis* cells were induced to express the fusion protein at 37°C, grown to OD_{600} = 0.50, harvested, lysed and centrifuged to obtain crude extract, which was ultracentrifuged at 100,000 × g to obtain membrane pellet and supernatant. The pellet was resuspended in PBS buffer with the same volume as the supernatant and the same amount of pellet suspension and supernatant was used in fluorescence measurement with excitation at 488 nm and emission at 507 nm. The experiments were performed in triplicates and the fluorescence was normalized using the result for the wild-type protein. The wild type GFP_m-PlsX is 7.85 fold in cytosol more than in membrane.

.

Supplementary Figure 6. Catalytic activity of PlsX and its mutant K257A. a. SDS-PAGE gel of the purified PlsX and its K257A mutant. b. Normalized catalytic activity of the wild-type PlsX and its K257A mutant. See 'Materials and Methods' for the assay conditions of the enzymatic activity.

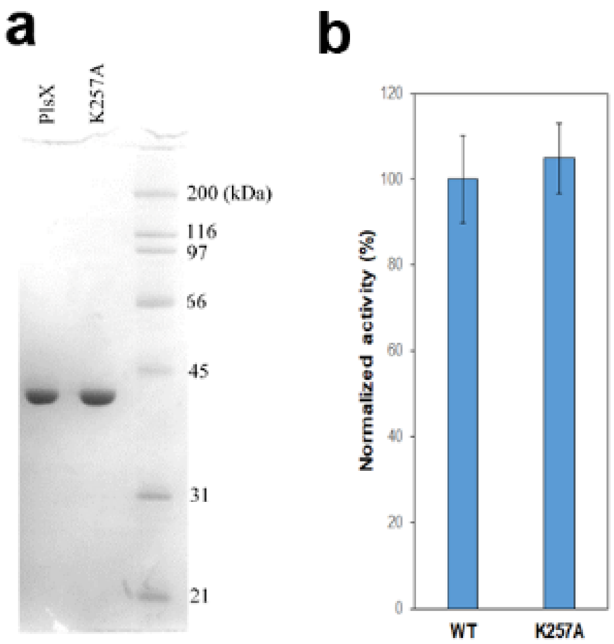

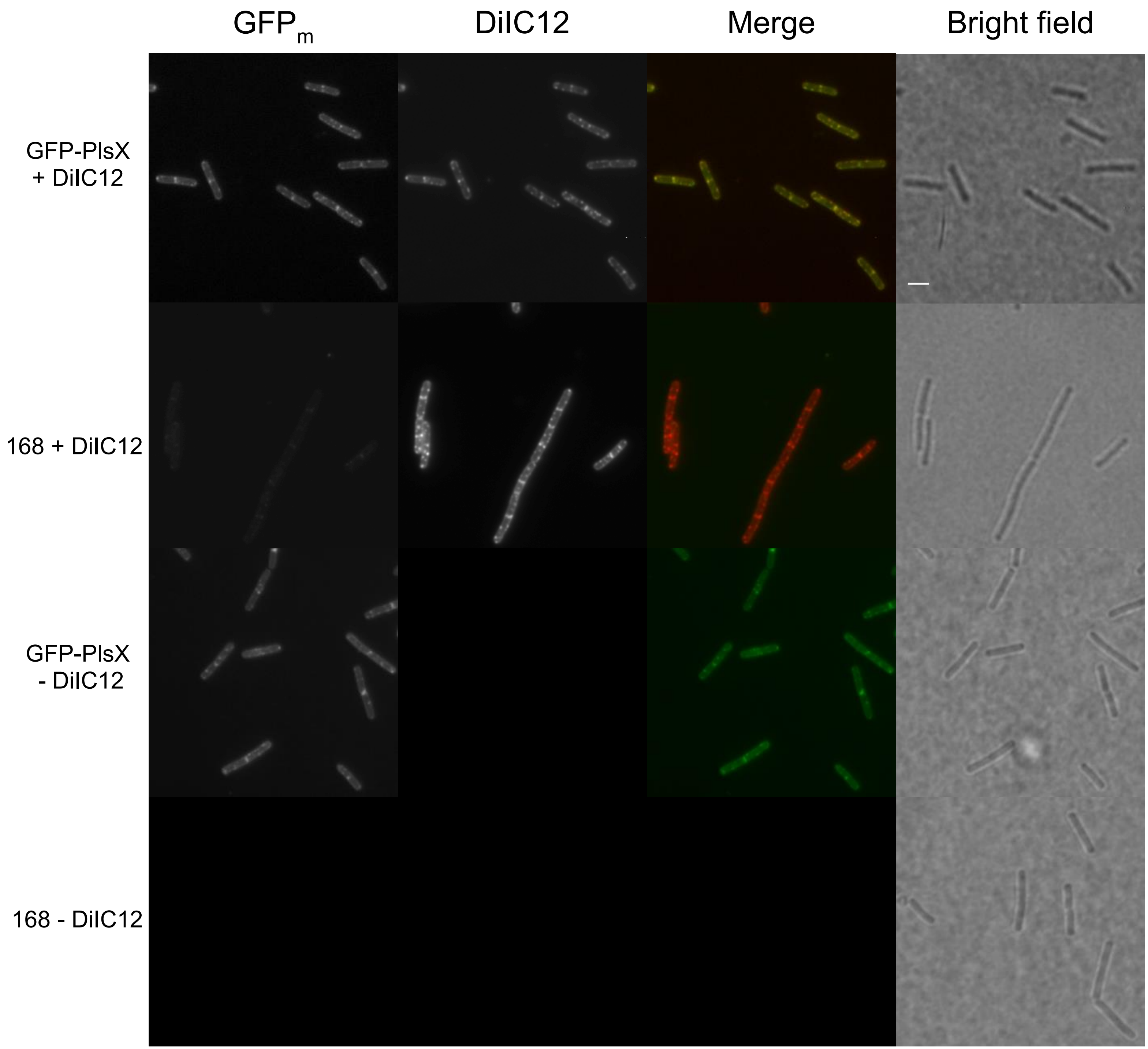

Supplementary Figure 7. Bleed-through test of the fluorescence microscopic imaging. The intensity of excitation light and settings for the camera were optimized to ensure no bleed-through as shown in the figure and used consistently in all imaging tasks. The test was conducted with *Bacillus subtilis* strain 168 (denoted by '168') with or without expression of GFP-PlsX from the ecotopic insertion site at the *amyE* locus. DiIC12 was used at a concentration of 2 µg/ml in the cell staining. The scale bar is 5 µm.

Supplementary Table 1. Deoxynucleotide primers used in this study.

Primer	Sequence
K257A-for	CGTCTACTTTGACATCCGCGCTTGCAGCAGCTGTGCTG
K257A-rev	CAGCACAGCTGCTGCAAGCGCGGATGTCAAAGTAGACG
S256A-for	GACGTCTACTTTGACAGCCAAGCTTGCAGCAGCTGTGC
S256A-rev	GCACAGCTGCTGCAAGCTTGGCTGTCAAAGTAGACGTC
T255A-for	CGTAATGACGTCTACTTTGGCATCCAAGCTTGCAGCAGC
T255A-rev	GCTGCTGCAAGCTTGGATGCCAAAGTAGACGTCATTACG
L254E-for	CGTAATGACGTCTACTGAGACATCCAAGCTTGCAGC
L254E-rev	GCTGCAAGCTTGGATGTCTCAGTAGACGTCATTACG
L258D-for	CTACTTTGACATCCAAGGATGCAGCAGCTGTGCTGAAACC
L258D-rev	GGTTTCAGCACAGCTGCTGCATCCTTGGATGTCAAAGTAG
A261D-for	CATCCAAGCTTGCAGCAGATGTGCTGAAACCAAAATTG
A261D-rev	CAATTTTGGTTTCAGCACATCTGCTGCAAGCTTGGATG
S252W-for	AGAGACGTAATGACGTGGACTTTGACATCCAAG
S252W-rev	CTTGGATGTCAAAGTCCACGTCATTACGTCTCT
T253W-for	AATGACGTCTTGGTTGACATCCAAGCTTGCAGCAGCTGTG
T253W-rev	TGGATGTCAACCAAGACGTCATTACGTCTCTCATCATTTTAAAAATTG
T255W-for	ATGACGTCTACTTTGTGGTCCAAGCTTGCAGCA
T255W-rev	TGCTGCAAGCTTGGACCACAAAGTAGACGTCAT
M250A-for	GATGAGAGACGTAGCGACGTCTACTTTGAC
M250A-rev	GTCAAAGTAGACGTCGCTACGTCTCTCATC
V262A-for	GCTTGCAGCAGCTGCGCTGAAACCAAAATTG
V262A-rev	CAATTTTGGTTTCAGCGCAGCTGCTGCAAGC
L254A-for	CGTAATGACGTCTACTGCGACATCCAAGCTTGC
L254A-rev	GCAAGCTTGGATGTCGCAGTAGACGTCATTACG
L258A-for	CTTTGACATCCAAGGCTGCAGCAGCTGTGCTG
L258A-rev	CAGCACAGCTGCTGCAGCCTTGGATGTCAAAG
T253A-for	CAAGCTTGGATGTCAAAGCAGACGTCATTACGTCTCT
T253A-rev	AGAGACGTAATGACGTCTGCTTTGACATCCAAGCTTG
L254W-for	CGTCTACTTGGACATCCAAGCTTGCAGCAGCTGTGCT
L254W-rev	TTGGATGTCCAAGTAGACGTCATTACGTCTCTCATCATTTTAAA
A261F-for	TTCAATTTTGGTTTCAGCACAAATGCTGCAAGCTTGGATGTCAAAG
A261F-rev	CTTTGACATCCAAGCTTGCAGCATTTGTGCTGAAACCAAAATTGAA
L254D-for	GCTGCTGCAAGCTTGGATGTATCAGTAGACGTCATTACGTCTCTCA
L254D-rev	TGAGAGACGTAATGACGTCTACTGATACATCCAAGCTTGCAGCAGC
L258S-for	TTTCAGCACAGCTGCTGCACTCTTGGATGTCAAAGTAGAC
L258S-rev	GTCTACTTTGACATCCAAGAGTGCAGCAGCTGTGCTGAAA
T251A-for	GATGAGAGACGTAATGGCGTCTACTTTGACATCC
T251A-rev	GGATGTCAAAGTAGACGCCATTACGTCTCTCATC
T251W-for	GATGAGAGACGTAATGTGGTCTACTTTGACATCC
T251W-rev	GGATGTCAAAGTAGACCACATTACGTCTCTCATC
A259T-for	GACATCCAAGCTTACAGCAGCTGTGCTGAAAC
A259T-rev	GTTTCAGCACAGCTGCTGTAAGCTTGGATGTC
A261W-for	CTTGCAGCATGGGTGCTGAAACCAAAATTGAAAGAAATGAAAATGAA
A261W-rev	TTTCAGCACCCATGCTGCAAGCTTGGATGTCAAAGTAGACG
A261L-for	CTTGCAGCACTTGTGCTGAAACCAAAATTGAAAGAAATGAAAATGAA

A261L-rev	TTTCAGCACAAGTGCTGCAAGCTTGGATGTCAAAGTAGACG
WT-for	TATTGGATCCTGATGAGAATAGCTGTAGATGC
WT-rev	TATTCTCGAGCTACTCATCTGTTTTTTCTT
GFP-for	CCTGTCCACACAATCTAAACTTTCGAAAGATCCC
GFP-rev	GGGATCTTTCGAAAGTTTAGATTGTGTGGACAGG
PlsX-for	TATTCCATGGGTATGAGAATAGCTGTAGATGC
PlsX-rev	TATTCTCGAGCTCATCTGTTTTTTCTTCTT
MT-for	TATTGGTACCATGAGAATAGCTGTAGATGC
MT-rev	TATTCTCGAGCTACTCATCTGTTTTTTCTT
HX-1	GATCCGTATGACGTCTACTTTGACATCCAAGCTTGCAGCAGCTGTGTAGC
HX-2	TCGAGCTACACAGCTGCTGCAAGCTTGGATGTCAAAGTAGACGTCATACG
