# Supplementary material for: Identification of an amphipathic peptide sensor of the Bacillus subtilis fluid membrane microdomains
Source: Commun Biol. 2019 Aug 20;2:316. doi: 10.1038/s42003-019-0562-8 (PMC6702220; doi:10.1038/s42003-019-0562-8)
Supplement: Supplementary file 2 — Description of additional supplementary files [file 42003_2019_562_MOESM2_ESM.docx]

Description of additional supplementary files

Supplementary Data 1.xls contains the source data underlying Figure 4.
