# Supplementary material for: Identification of an amphipathic peptide sensor of the Bacillus subtilis fluid membrane microdomains
Source: Commun Biol. 2019 Aug 20;2:316. doi: 10.1038/s42003-019-0562-8 (PMC6702220; doi:10.1038/s42003-019-0562-8)
Supplement: Supplementary file 4 — Reporting Summary [file 42003_2019_562_MOESM4_ESM.pdf]

nature research

Corresponding author(s): Zhihong Guo

Last updated by author(s): Jul 19, 2019

Reporting Summary

Nature Research wishes to improve the reproducibility of the work that we publish. This form provides structure for consistency and transparency in reporting. For further information on Nature Research policies, see Authors & Referees and the Editorial Policy Checklist.

Statistics

For all statistical analyses, confirm that the following items are present in the figure legend, table legend, main text, or Methods section.

n/a	Confirmed	
☐	☒	The exact sample size (*n*) for each experimental group/condition, given as a discrete number and unit of measurement
☐	☒	A statement on whether measurements were taken from distinct samples or whether the same sample was measured repeatedly
☐	☒	The statistical test(s) used AND whether they are one- or two-sided<br>*Only common tests should be described solely by name; describe more complex techniques in the Methods section.*
☐	☒	A description of all covariates tested
☒	☐	A description of any assumptions or corrections, such as tests of normality and adjustment for multiple comparisons
☐	☒	A full description of the statistical parameters including central tendency (e.g. means) or other basic estimates (e.g. regression coefficient) AND variation (e.g. standard deviation) or associated estimates of uncertainty (e.g. confidence intervals)
☒	☐	For null hypothesis testing, the test statistic (e.g. *F*, *t*, *r*) with confidence intervals, effect sizes, degrees of freedom and *P* value noted<br>*Give P values as exact values whenever suitable.*
☒	☐	For Bayesian analysis, information on the choice of priors and Markov chain Monte Carlo settings
☒	☐	For hierarchical and complex designs, identification of the appropriate level for tests and full reporting of outcomes
☐	☒	Estimates of effect sizes (e.g. Cohen's *d*, Pearson's *r*), indicating how they were calculated

Our web collection on statistics for biologists contains articles on many of the points above.

Software and code

Policy information about availability of computer code

Data collection	Not applicable
Data analysis	Not applicable

For manuscripts utilizing custom algorithms or software that are central to the research but not yet described in published literature, software must be made available to editors/reviewers. We strongly encourage code deposition in a community repository (e.g. GitHub). See the Nature Research guidelines for submitting code & software for further information.

Data

Policy information about availability of data

All manuscripts must include a data availability statement. This statement should provide the following information, where applicable:

- Accession codes, unique identifiers, or web links for publicly available datasets
- A list of figures that have associated raw data
- A description of any restrictions on data availability

The reported crystal structure has been deposited in wwPDB with the following accession code: 6A1K. Raw data giving rise to Figures 2 and 3 are available upon request.

Field-specific reporting

Please select the one below that is the best fit for your research. If you are not sure, read the appropriate sections before making your selection.

☒ Life sciences ☐ Behavioural & social sciences ☐ Ecological, evolutionary & environmental sciences

October 2018

Life sciences study design

All studies must disclose on these points even when the disclosure is negative.

Sample size	In Figure 2 and Figure 3, only a few cells were shown to represent a much larger number of cells, of which at least 10 cells from a larger region were used to calculate the reported Rr value as shown in the supplementary Figure 3. The raw images, from which the presented images were cut and prepared, are available upon request.
Data exclusions	No data were excluded.
Replication	All the imaging experiments were repeated at least once at a different time or by a different researcher. All the reported images and data were successfully reproduced.
Randomization	The participants were randomly assigned to construct expression plasmids for the PlsX mutants, express them and image them for subcellular localization, with the first author doing most of the work.
Blinding	Imaging of each cell strain and analysis of the images were performed independently by each investigator.

Reporting for specific materials, systems and methods

We require information from authors about some types of materials, experimental systems and methods used in many studies. Here, indicate whether each material, system or method listed is relevant to your study. If you are not sure if a list item applies to your research, read the appropriate section before selecting a response.

Materials & experimental systems

n/a	Involved in the study	
☐	☒	Antibodies
☒	☐	Eukaryotic cell lines
☒	☐	Palaeontology
☒	☐	Animals and other organisms
☒	☐	Human research participants
☒	☐	Clinical data

Methods

n/a	Involved in the study	
☒	☐	ChIP-seq
☒	☐	Flow cytometry
☒	☐	MRI-based neuroimaging

Antibodies

Antibodies used	GFP-specific monoclonal antibody from mouse (ThermoFisher) and the HRP-goat anti-mouse IgG antibody (ThermoFisher)
Validation	The manufacturer's statement (https://assets.thermofisher.com/TFS-Assets/LSG/manuals/33-2600_Mouse%20anti-GFP%20%20Rev%200809.pdf): This monoclonal antibody is specific for the green fluorescent protein (GFP) from the jellyfish Aequorea victoria. This antibody can be used to detect GFP and GFP variants in Western blots and produces a highly specific signal with very low background. On Western blots, the antibody can easily detect as little as 40 pg of recombinant GFP protein. Lower amounts of protein can be detected if chemiluminescent detection is employed.<br><br>This antibody was validated to produce a specific signal for GFP or its fusion proteins expressed in B. subtilis as shown by the controls in the Supplementary Figure 2.
